# External validation of a top-ranked model from the RSNA pulmonary embolism detection challenge: assessment of generalizability

**DOI:** 10.1038/s41598-026-65551-z

**Published:** 2026-08-08

**Authors:** Patricia Hahlbohm, Sebastian Starke, Felix Schön, Leo Misera, Marius Erdmann, Ralf-Thorsten Hoffmann, Alex Zwanenburg, Jens-Peter Kühn, Steffen Löck

**Affiliations:** 1 Institute and Polyclinic for Diagnostic and Interventional Radiology, Faculty of Medicine and University Hospital Carl Gustav Carus Dresden, TUD Dresden University of Technology, Fetscherstraße 74, 01307 Dresden, Germany; 2 Helmholtz AI / Helmholtz-Zentrum Dresden-Rossendorf, Department of Information Services and Computing, Dresden, Germany; 3 Else Kroener Fresenius Center for Digital Health, TU Dresden, Dresden, Germany; 4 OncoRay – National Center for Radiation Research in Oncology, Faculty of Medicine and University Hospital Carl Gustav Carus Dresden, TU Dresden and Helmholtz-Zentrum Dresden-Rossendorf, Dresden, Germany; 5 National Center for Tumor Diseases (NCT), NCT/UCC Dresden, a partnership between DKFZ, Faculty of Medicine and University Hospital Carl Gustav Carus Dresden, TU Dresden and Helmholtz-Zentrum Dresden-Rossendorf (HZDR), Dresden, Germany; 6 Department of Radiotherapy and Radiation Oncology, Faculty of Medicine and University Hospital Carl Gustav Carus Dresden, TU Dresden, Dresden, Germany; 7 German Cancer Consortium (DKTK), Partner Site Dresden, and German Cancer Research Center (DKFZ), Heidelberg, Germany; 8 Helmholtz-Zentrum Dresden-Rossendorf, Institute of Radiooncology – OncoRay, Dresden, Germany

**Keywords:** Deep learning, External validation, Pulmonary embolism, Diseases, Health care, Medical research

## Abstract

To externally validate the RSNA 2020 challenge 2^nd^-place deep-learning (DL) algorithm for detecting pulmonary embolism (PE) on computed tomography pulmonary angiography (CTPA) scans with additional focus on subsegmental-only PEs (SSPE). Between 2015 and 2022 1,038 CTPAs were retrospectively enrolled. An experienced radiologist (> five years) labelled CTPAs for the presence, location (central, lobar and segmental, subsegmental-only) and side (right-sided, left-sided, bilateral) of PE. The DL model was tested for its ability to predict these labels by analyzing accuracy, sensitivity, specificity and area under the receiver operating characteristic curve (AUROC) using different model outcome probabilities for each analysis. Overall, 136 central, 375 peripheral PEs (303 lobar and segmental PEs, 72 SSPEs), and 527 patients without any PE were analyzed. The model correctly predicted the presence of any PE in 921/1,038 patients (88.7%), yielding a sensitivity of 0.80, specificity of 0.97 and AUROC of 0.94. No central PE was missed, whereas 100/375 (26.7%) peripheral PEs remained undetected. Using the corresponding model output probability, the central PE status was correctly identified in 999/1,038 (96.2%) patients, while peripheral PE was correctly identified in 785/1,038 (75.6%) patients. This corresponded to sensitivities of 0.94 and 0.77, specificities of 0.97 and 0.75, and AUROC values of 0.99 and 0.74, respectively. The model performed better in detecting right-sided compared to left-sided PEs (AUROC: right- vs. left-sided: 0.95 and 0.92, *p* < 0.05). SSPE status was correctly predicted in 796/1,038 (76.7%) patients, yielding a sensitivity of 0.94, a specificity of 0.22, and an AUROC of 0.48, respectively. The model demonstrated high performance in detecting any PE, performing best on central and slightly lower on lobar and segmental PE. Predicting the presence of SSPEs was difficult, likely due to the model not being explicitly trained for this subtype.

**Supplementary Information:**

The online version contains supplementary material available at 10.1038/s41598-026-65551-z.

## Introduction

Pulmonary embolism (PE) is a potentially life-threatening disease, which causes approximately up to 300,000 deaths in the United States per year. Early diagnosis following prompt treatment could improve the overall patient´s outcome^1,2^.

Computed tomographic pulmonary angiography (CTPA) is the gold-standard for diagnosing clinical suspected PE with high diagnostic accuracy and is therefore routinely used in clinical care^2–4^. Due to continuous technological advancements, deep learning (DL) algorithms have increasingly become the focus of scientific efforts in recent years, also demonstrating promising results in PE diagnosis. In particular, they have been observed to enhance PE detection accuracy in CTPA, which was among others shown by a meta-analysis reporting a pooled sensitivity of 0.88 and specificity of 0.86, respectively^4^. However, their integration into daily clinical practice remains challenging due to limited acceptance and understanding of the technology^5^.

In 2020, the Radiological Society of North America (RSNA) hosted a competition aimed at developing DL algorithms for detecting PE on CTPA scans^6^. A publicly available dataset of 9,446 CTPA scans was provided for the competition, containing multiple labels on study level, such as whether the study was PE positive, the location of the PE (central, left- or right-sided), and the ratio between the right and left ventricle of the heart (RV/LV). Furthermore one binary label (PE present or not) was provided for each slice^7^. As per the competition rules, the participating solutions were made publicly available and included the programming code.

However, external validation of the solutions was not part of the competition and is therefore lacking. A recent review found that 81% of the analyzed algorithms showed decreased performance upon external validation, with nearly 25% reporting a substantial drop (≥ 0.1 in area under receiver operating characteristic curve (AUROC))^8^. External validation of DL models is crucial in the process of clinical implementation. Roberts et al. emphasized that a sufficient external validation is an important step for ensuring a model’s generalizability to other cohorts^9^.

The focus of the RSNA PE challenge was on diagnosing central and peripheral PEs down to the segmental level of the pulmonary arteries. Evaluation of DL-aided detection of subsegmental-only PEs (SSPE) is currently lacking. Although the clinical relevance of SSPEs is under discussion, accurate diagnosis is crucial in clinical care as SSPEs are associated with a higher recurrence rate compared to other PE types^2,10^. However, SSPEs are often considered challenging to detect, underscoring the need for further development, particularly in DL-based algorithms.

Since the RSNA PE challenge winner’s solution has already been externally validated for the general PE prediction by Langius-Wiffen et al.^3^, the aim of the present study was to carry out a more extensive external validation of the 2^nd^-place DL model of Pan^11^ with respect to most outcome parameters as well as an evaluation of its performance on SSPEs.

## Materials and methods

### Patient inclusion and reference standard

This study was approved by the local ethics committee at the Technical University of Dresden (EK 349082023) and conforms to the Declaration of Helsinki. The informed consent was waived by the ethics committee due to the retrospective nature of the study.

All patients who received a CTPA scan in our department between 2015 and 2022 were retrospectively enrolled. Exclusion criteria were an age younger than 18 years or if the CT scan was a follow-up scan of the same patient. The local radiology information system was searched for the corresponding reports, describing whether a PE was present or not. All radiology reports, which were either written by a board-certified radiologist with more than 5 years of experience or by a resident and approved by a board-certified radiologist, were screened for the first labelling. Studies with ambiguous radiology reports that did not clearly state the presence or absence of a PE, were excluded. All studies positively marked for the presence of PE were double-checked by a radiologist with 5 years of experience in thoracic imaging (F.S.) and finally labeled as central, lobar and segmental or SSPE. Labelling was done according to the RSNA pulmonary embolism dataset definition^7^. Thus, a study was labeled as central, when the most proximal PE was in the right or left main pulmonary artery up to the first arterial branch. A more distal embolism was considered as lobar and segmental PE, which also corresponds to the differentiation used by Leonhardi et al.^12^. If the study contained a central PE that extended up to the periphery, it was labeled as central as well. Likewise, studies containing lobar or segmental PEs that extended to subsegmental levels were labeled as lobar and segmental. Embolisms on purely subsegmental level were labeled as SSPEs. The peripheral PE categorization includes lobar, segmental and SSPE. Furthermore, the side (right/left/bilateral) of PE was added. A second reader with more than 15 years of experience as a board-certified radiologist (J.P.K.) reviewed the studies initially labeled as SSPE. Differences between the first and second reader were solved within a consensus meeting. Additionally, thirty randomly selected studies initially labeled as negative for PE were also re-checked by the radiologist (F.S.) to verify the accuracy of the report.

**Fig. 1:**
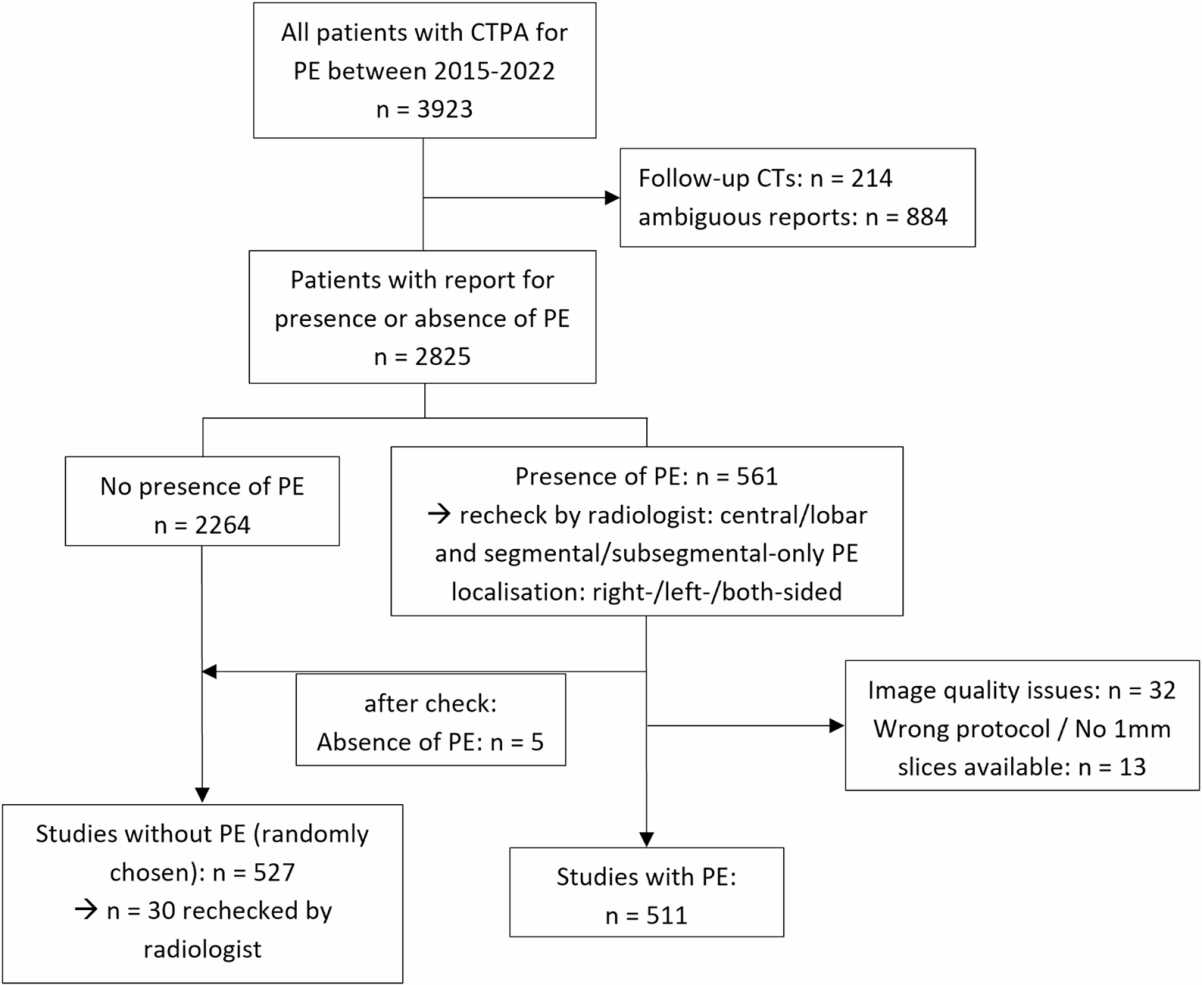
Patient inclusion scheme.

The inclusion workflow can be seen in Fig. 1. Finally, the images were exported from the local picture archiving system (Impax EE R20, Agfa Healthcare, Mortsel, Belgium).

### CT scanning protocol

All CT scans were acquired in clinical routine using two 128-slice CT scanners (Siemens Somatom Definition Edge and AS+, Siemens Healthineers, Erlangen, Germany). CTPA scans were acquired as a bolus-triggered protocol with a threshold of 100 HU, given approximately 60 ml contrast agent (Ultravist 370, Bayer Vital GmbH, Leverkusen, Germany) with a flow of 3–4 ml/s. Dose saving options for automatic dose control (CareDose4D, Siemens Healthineers, Erlangen, Germany) and automatic tube voltage selection (CarekV, Siemens Healthineers, Erlangen, Germany) were used. All scans were reconstructed with a slice thickness of 1 and 3 mm using a soft tissue kernel, and in some studies additionally using iterative reconstruction (SAFIRE/ADMIRE level 3, Siemens Healthineers, Erlangen, Germany). For this study, only scans with 1 mm slices and a slice increment of 0.8 mm were considered.

### Deep learning model for detecting PE

The model by Pan^11^ is a collection of different underlying models, consisting of two feature extractors, twelve transformers, five time-distributed convolutional neural networks (CNN), one heart section classifier, five RV/LV ratio 3D feature extractors, and five RV/LV ratio linear models.

For this study the model components not corresponding to cardiac assessments were validated. First, a feature-extracting CNN based on the ResNeSt-50 architecture calculates primary image- and examination-level labels (without RV/LV predictions). Second, predictions were further improved using sequence modeling in terms of a slice and an exam transformer. Finally, a time-distributed CNN was used to predict the final examination-level PE labels. A detailed description of the model architecture, training process, and components can be found in the corresponding paper^11^ and GitHub Repository^13^. No image preprocessing was necessary, before the CT studies were fed into the DL model.

### Statistical analysis

To evaluate the model’s performance, the AUROC with a 95% confidence interval (CI) calculated as an empirical bootstrap (*n* = 1000) estimate and a p-value calculated as an empirical one-sample two-sided location test^14^ with H0: AUROC = 0.5 were determined. Additionally, the accuracy, a confusion matrix, sensitivity and specificity were calculated for every prediction task after maximizing the Youden index to identify a threshold for an optimal model selectivity. For sensitivity and specificity, 95% CIs were calculated analogously to the AUROC-CI by using an empirical bootstrap approach. The threshold was held constant across all bootstrap samples.

To evaluate the influence of PE prevalence on the model’s performance for detecting the presence of any PE, the dataset was subsampled. All PE negative studies were kept. PE positive studies were randomly selected (without replacement) to simulate overall prevalence of PE of 5% to 25%, in 5% steps. The process was repeated 1000 times for each prevalence level.

For comparison of the model performance predicting right-sided (and bilateral) or left-sided (and bilateral) PE, the p-value regarding the AUROC comparison was determined as an empirical two-sample two-sided location test^14^. Because the RSNA PE challenge involved predicting peripheral PE for each side instead of the overall presence of a peripheral PE, the higher predicted probability of either left- or right-sided PE was used during validation.

Furthermore, the model did not provide a direct prediction of SSPEs. Therefore, in order to determine whether a prediction of the presence of SSPE was possible from the provided model output probabilities, we used the probability of the presence of PE and additionally calculated the balanced accuracy. As part of a sensitivity analysis, different values for lower and upper thresholds (with 0.05 increments) were exploratively tested and the sensitivity, specificity, a confusion matrix and balanced accuracy metrics were calculated.

An occlusion-based sensitivity analysis was performed by sliding a fixed-size occlusion window across the CT scans. At each position, the occluded image was forwarded through the model. Changes in the predicted probability for the presence of PE were plotted.

Furthermore, we investigated whether a combination of multiple model output parameters could improve the prediction (AUROC) of the binary or multinomial diagnostic findings. Binary findings were: presence of PE, central PE, right- and left-sided PE (all direct model outputs), presence of a peripheral PE and SSPE. Multinomial findings were: the exact prediction of the PE diagnosis (central vs. lobar and segmental vs. SSPE vs. no PE) and the side of PE (left vs. right vs. bilateral). For all binary findings, a binary logistic regression model was used, whereas for all multinomial findings a multinomial logistic regression model was used. The entire dataset was split into a training and test set using a 60:40 split. All models were optimized using the training set with respect to the AUROC using forward feature selection in an internal 5-fold cross validation experiment. The AUROC values were calculated on the test dataset. For multinomial findings, the AUROC was calculated using the one-versus-rest approach. Forward selection was stopped when the AUROC did not improve by more than 0.01 when adding another feature.

Calculations were performed using Python Version 3.12.7 with its specific libraries (e.g. scikit-learn version 1.5.1). The significance level was set to α = 0.05.

## Results

### Patient characteristics

After checking all cases initially marked positive for PE (*n* = 561) and randomly chosen negatively marked cases (*n* = 527, of whom *n* = 30 were rechecked), the dataset contained 1038 patients (mean age 68.0 ± 16.0 years, 595 men), see Fig. 1. Central PE was diagnosed in 136 patients (13.1%), 303 patients (29.2%) had a lobar and segmental PE. 72 patients (6.9%) were initially labeled with SSPE. Thus, there were 375 patients with peripheral PE. The second reading confirmed SSPE status in approximately 96% (69 / 72 SSPEs). The three discrepancies were solved, resulting in 72 SSPEs (Table 1).

**Table 1:** Patient demographics.

Patient demographics	Number (*n* = 1038)
**Gender**	
Male, n (%)	595 (57.3)
Female, n (%)	443 (42.7)
**Mean age ± SD (range), years**	68.0 ± 16.0 (19.0–98.0)
**Diagnostic findings:**	
PE present, n (%)	511 (49.2)
No PE present, n (%)	527 (50.8)
*Location of PE:*	
Central PE, n (%)	136 (13.1)
Lobar and segmental PE, n (%)	303 (29.2)
Subsegmental-only PE, n (%)	72 (6.9)
No PE, n (%)	527 (50.8)
*Side of PE:*	
Left-sided, n (%)	55 (5.3)
Right-sided, n (%)	146 (14.1)
Bilateral, n (%)	309 (29.8)
No PE, n (%)	527 (50.8)

*PE* pulmonary embolism, *SD* standard deviation.

### Evaluation of direct model output parameters

**Fig. 2:**
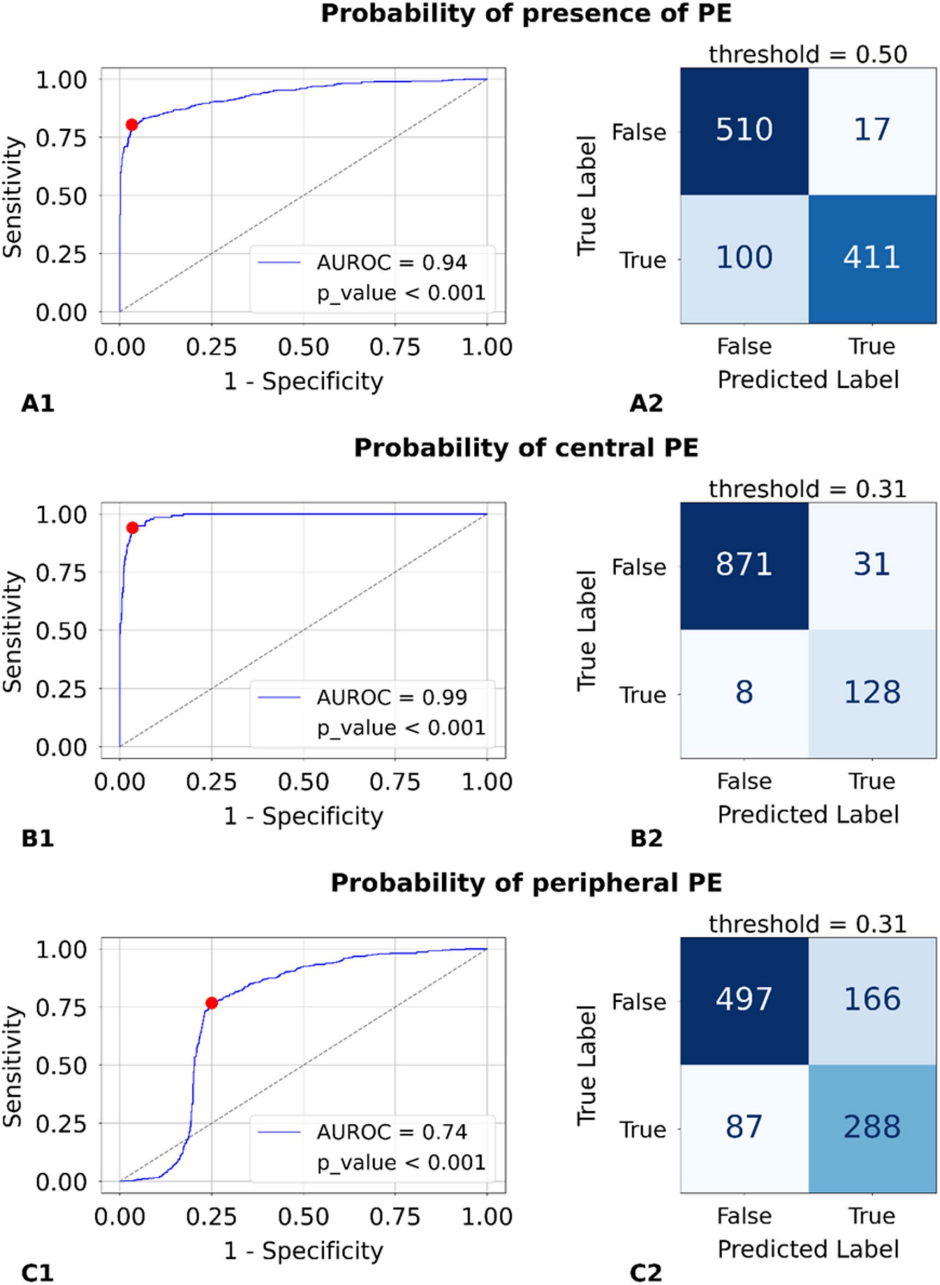
AUROC curves (left) and confusion matrices (right) for the prediction of the labels for the presence of a PE, a central PE and a peripheral PE based on the model output probabilities. The threshold for the confusion matrix was determined by calculating the maximum Youden index, which is visualized by the red dot.

**Fig. 3:**
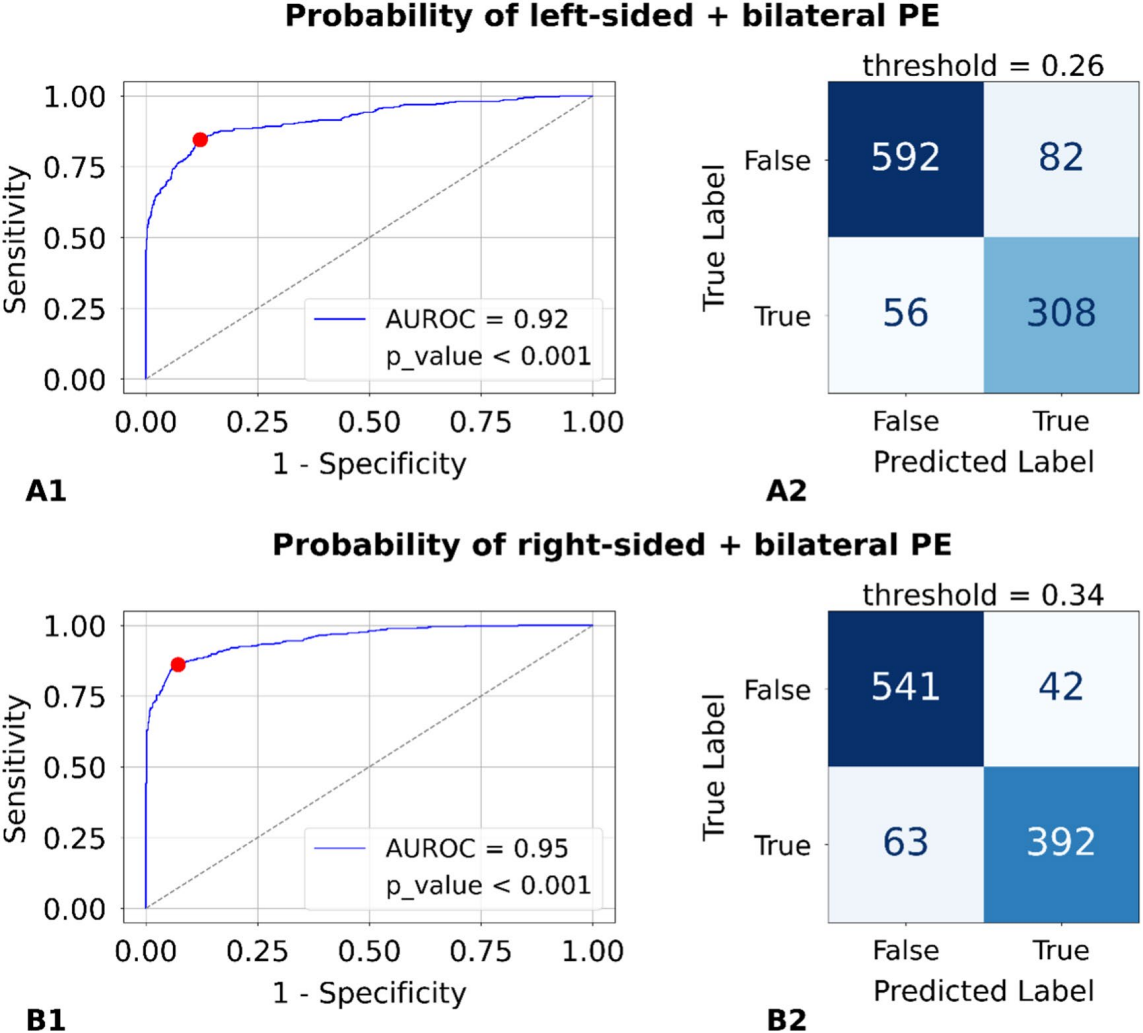
AUROC curves (left) and confusion matrices (right) for the prediction of the labels for the side of the PE considering one-sided and bilateral PEs based on the model output probabilities. The threshold for the confusion matrix was determined by calculating the maximum Youden index, which is visualized by the red dot.

First, we validated the four direct model output probabilities for the presence of any PE, the presence of a central PE, the presence of a right- or left-sided PE and the derived probability for a peripheral PE. Therefore, our class labels were binarized to match the model output. Resulting model performance including the threshold for the confusion matrix can be seen in Table 2, AUROC curves and confusion matrices are shown in Figs. 2 and 3, respectively.

**Fig. 4:**
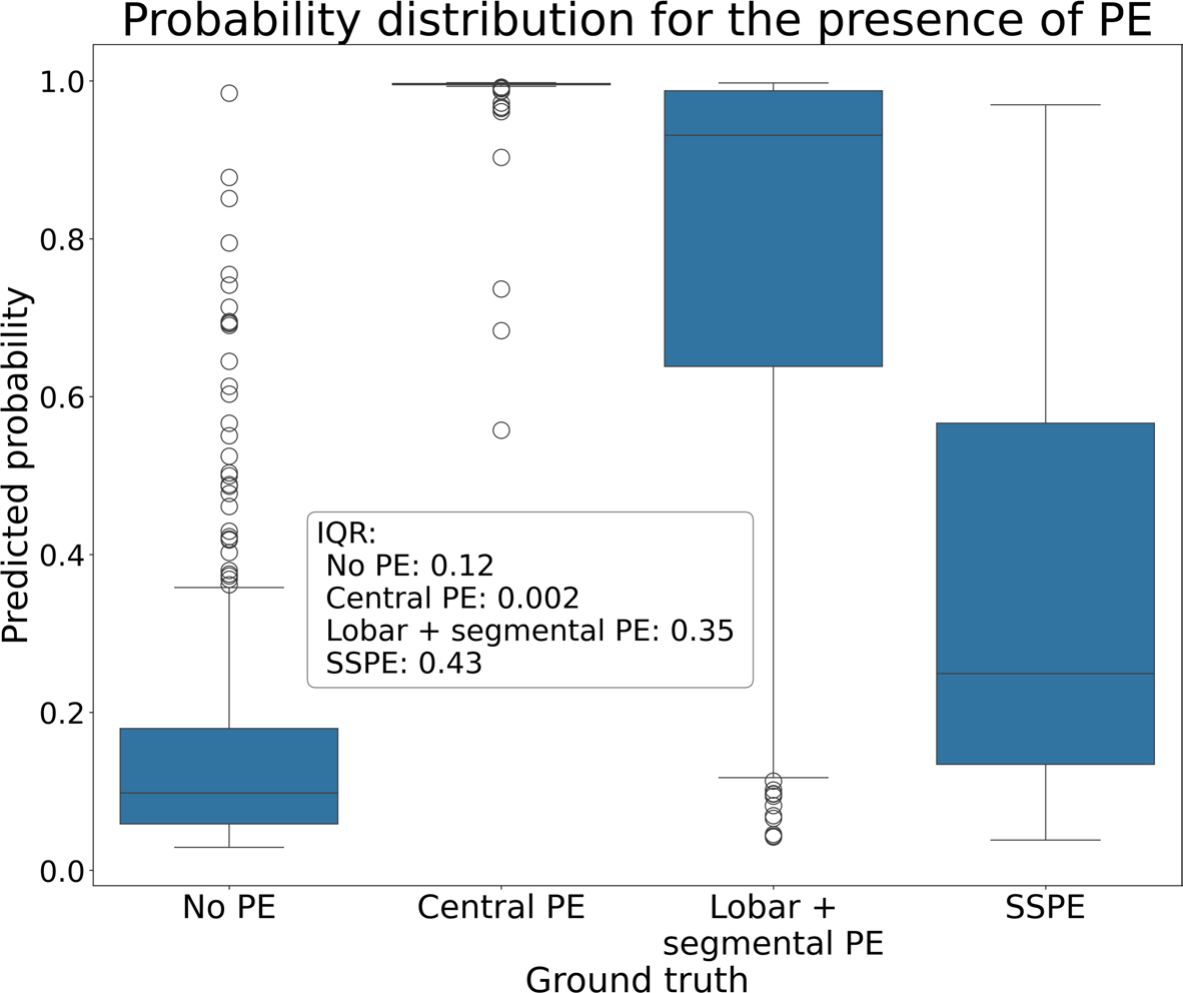
Probability distribution for a presence of a PE within a study for each class label together with the interquartile range (IQR).

The model correctly predicted the presence of any PE in 921/1,038 (88.7%) cases, resulting in a sensitivity and specificity of 0.80 and 0.97, respectively, as well as an AUROC of 0.94 (CI: 0.92–0.95). The probability distribution, grouped by observed label, is shown in Fig. 4. The interquartile range (IQR) was low for studies without PE (IQR: 0.12) and central PE (IQR: 0.002) and higher for lobar and segmental (IQR: 0.35) and SSPE (IQR: 0.43).

Of the studies with observed PE, 411/511 (80.4%) were correctly classified, whereas 100/511 (19.6%) were incorrectly classified as not having any PE. Of these 100 false negative studies, no studies with central PE were missed, while 55 lobar and segmental and 45 SSPEs were not detected.

**Fig. 5:**
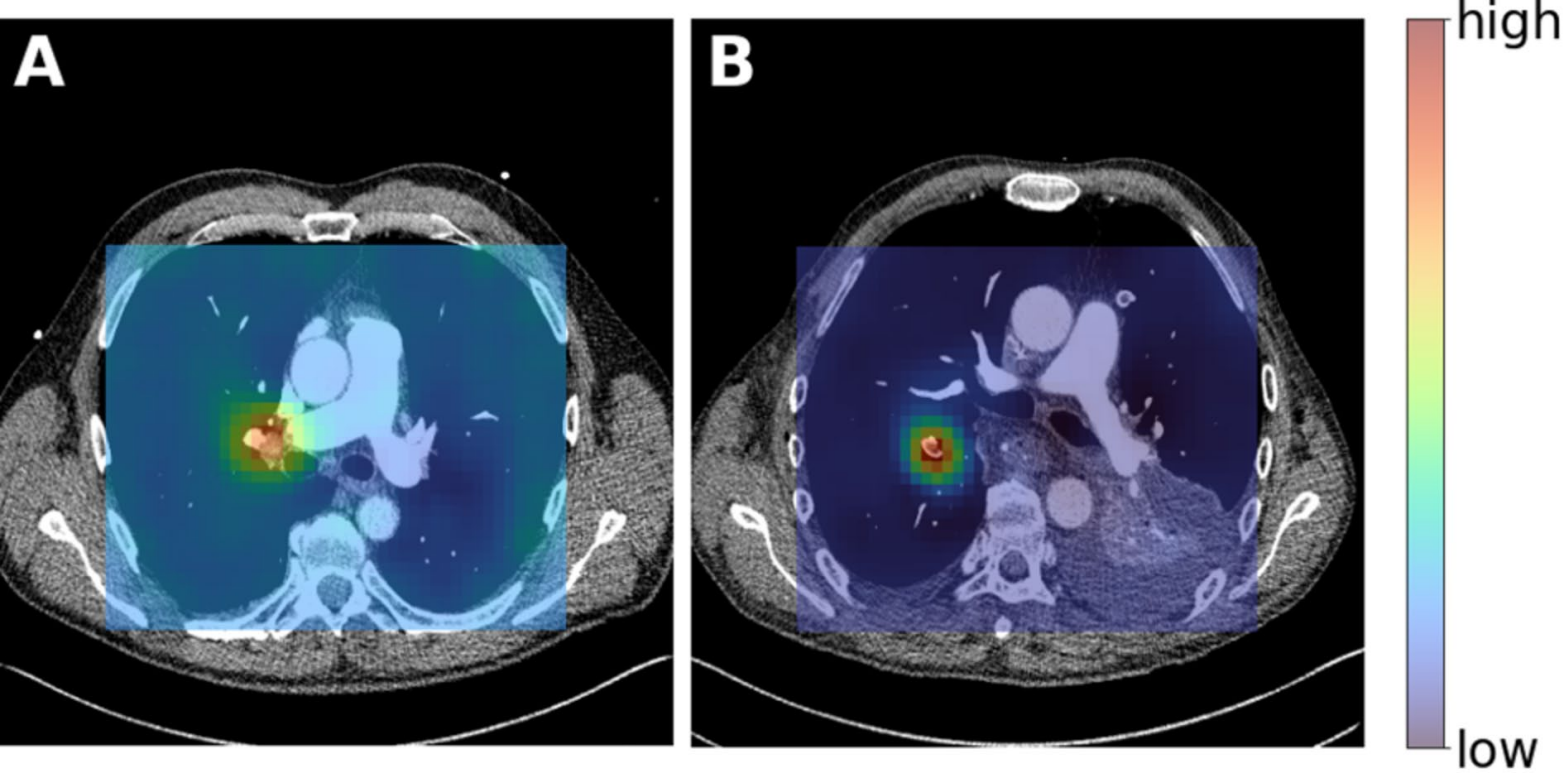
Occlusion maps generated using the deep-learning model output probability for the presence of a PE. Images show an example for a correctly identified study with a central PE (A) and a false positive study, where the model identified an inserted chest drain as PE (B).

Of the studies without observed PE, 17/527 (3.2%) studies were incorrectly classified as PE present with a probability of higher than 50%, and 5/17 (29.4%) had a presence probability even higher than 75%. In these 17 false positive cases, the predicted probability of a central PE was relatively low (< 26%), but the predicted probability of a peripheral PE was at least 50%. Concerning the five studies with a high probability of the presence of a PE, the probability of a peripheral PE was at least 69.0%. Rechecking these 17 studies showed that five studies with lobar and segmental or SSPE were missed during the initial labelling process, two studies remained unclear concerning the presence of an SSPE, and the remaining ten studies were wrongly classified by the model. Of the five studies that yielded a high probability for the presence of any PE (75%), three were wrongly classified by the model, and two lobar and segmental PEs were missed during initial labelling. Figure 5 shows occlusion maps for a study with a true positive finding and a different study where the finding was a false positive.

The model’s performance for detecting the presence of PE for different PE prevalences is shown in Table S1. Sensitivity and accuracy improved with lower prevalences (5%: 0.84 vs. 50%: 0.80 and 5%: 94.1% vs. 50%: 88.7%, respectively). Specificity decreased slightly with increasing prevalence (5%: 0.95 vs. 50% 0.97). CIs for all parameters narrowed with increasing prevalence, whereas AUROC values were highly similar.

The validation of the model’s predicted probability for central PE yielded a sensitivity of 0.94, a specificity of 0.97, and an AUROC of 0.99 (CI: 0.98–0.99). Eight central PEs were missed at the threshold corresponding to the maximum Youden index; however, all had a probability for the presence of a PE exceeding 55%, suggesting they would not have been overlooked when considering overall PE study probability. Additionally, each of these cases had at least one side (right or left) with a probability above 50%, and in four cases, both sides exceeded 80%. Among the 31 cases falsely classified as central PE, all were PE-positive, with 30 labeled as lobar and segmental and one as subsegmental-only. The corresponding study probability for the presence of a PE was > 92% for lobar and segmental PEs and 87% for the subsegmental-only case. For the 30 lobar and segmental studies, the mean probability for a central PE was 0.45 (range: 0.31–0.66), while the probability for a peripheral PE was substantially higher (mean: 0.96, range: 0.84–0.99). Thus, this “misclassification” resulted from a differing assessment of central vs. lobar and segmental PE.

For predicting any peripheral PE *(*lobar, segmental PE and SSPE), the higher model probability of either left- or right-sided PE was used. This approach resulted in a sensitivity of 0.77, a specificity of 0.75 and an AUROC of 0.74 (CI: 0.71–0.77). Subsequent rechecking of the 166 false positive studies, showed that 136/166 (81.9%) were studies labeled as central PE. Since central PE often extend to the periphery, a positive model prediction for peripheral PE may be in fact accurate. Of the remaining 30/166 (19.1%) studies, two were wrongly classified during the initial labelling process, two PEs were not described in the radiological report, and one was identified as a chronic PE. The remaining 25 studies were confirmed to be false positive studies, with 11/25 (44.0%) found to have artifacts or compression signs. The model mean probability for peripheral PE within these 25 cases was 0.50 (min – max: 0.32–0.79), the overall mean study probability for the presence of a PE was 0.56 (min – max: 0.24–0.85).

For the analysis of the probability for each side the respective single-sided PEs as well as all bilateral PEs were considered as true positive. For all four statistical parameters, the analysis of right-sided and bilateral PEs showed slightly better performance compared to left-sided and bilateral PEs (sensitivity: 0.86 vs. 0.85; specificity: 0.93 vs. 0.88; AUROC: 0.95 vs. 0.92 and accuracy: 89.9% vs. 86.7%).

**Table 2:** Results of the prediction of direct model parameters using area under the receiver operating characteristic curves (AUROC), sensitivity, specificity and accuracy.

Prediction label	Ground truth label	AUROC (95% CI)	*P*-value	Threshold	Sensitivity (95% CI)	Specificity (95% CI)	Accuracy [%]
PE present	PE Present	0.94 (0.92–0.95)	< 0.001	0.50	0.80 (0.77–0.84)	0.97 (0.95–0.98)	88.7
Central PE	Central PE	0.99 (0.98–0.99)	< 0.001	0.31	0.94 (0.90–0.98)	0.97 (0.95–0.98)	96.2
Peripheral PE*	Peripheral PE (Lobar + Segmental + SSPE)	0.74 (0.71–0.77)	< 0.001	0.31	0.77 (0.72–0.81)	0.75 (0.72–0.78)	75.6
Left-sided PE	Side of PE = left-sided or bilateral	0.92 (0.90–0.94)	< 0.001	0.26	0.85 (0.81–0.88)	0.88 (0.85–0.90)	86.7
Right-sided PE	Side of PE = right-sided or bilateral	0.95 (0.94–0.96)	< 0.001	0.34	0.86 (0.83–0.89)	0.93 (0.91–0.95)	89.9

*Peripheral PE ≙ maximum probability of left- or right-sided PE.

We investigated whether a combination of multiple model outcome probabilities as features for a logistic regression model could improve the performance (AUROC). The results are shown in Table 3. 4/6 (66.7%) of the binary prediction tasks could be predicted with an AUROC value ≥ 0.93 using a single model output probability. The selected features for these four tasks matched the corresponding model output, except in one case where the probability for a peripheral PE was used to predict the overall presence of PE within a study. Predicting a peripheral or SSPE by combining multiple features led to an increase in AUROC compared to using only one feature (peripheral: 0.89 vs. 0.74 and subsegmental-only: 0.69 vs. 0.48). The multinomial prediction tasks for determining the exact diagnosis and the side of PE shown in Table 3 benefitted (AUROC > 0.89) from combining three features.

**Table 3:** Results of combining one or multiple model outputs for several predicition tasks. The AUROC values were calculated on the test set.

Prediction tasks	Classification type of prediction	Number of used features	Selected feature(s)	AUROC (test set)
PE present	Binary	1	Peripheral PE	0.937
Central PE		1	Central PE	0.995
Peripheral PE		3	Negative exam for PE RV/LV Ratio < 1 Central PE	0.889
SSPE		2	Central PE Age	0.689
Left-sided PE		1	Left-sided PE	0.928
Right-sided PE		1	Right-sided PE	0.952
Exact PE Diagnosis (central, lobar and segmental, subsegmental-only, no PE)	multinomial	3	Peripheral PE Left-sided PE Central PE	0.892
Side with PE (right, left, bilateral)		3	Peripheral PE Right-sided PE Left-sided PE	0.897

### Evaluation of indirect model outputs

**Fig. 6:**
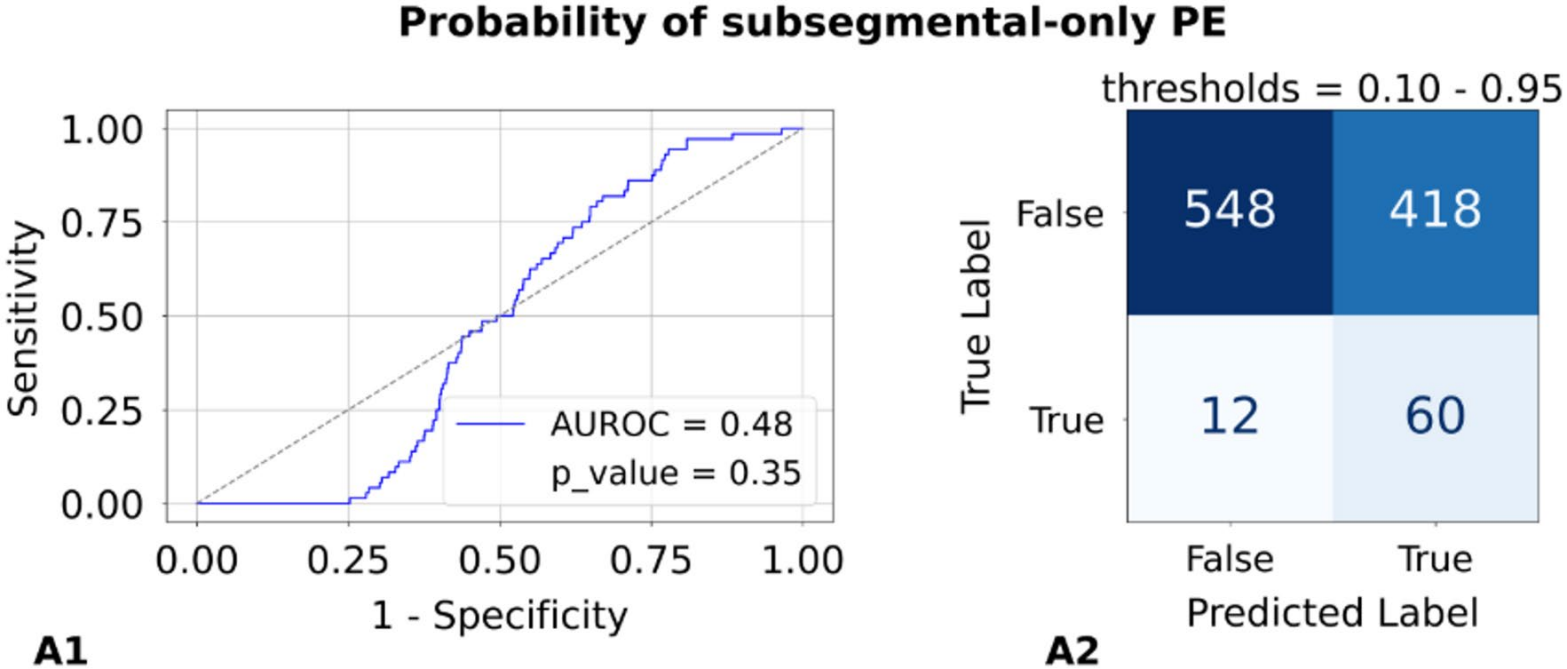
AUROC curve (left) and confusion matrix (right) for the prediction of a subsegmental-only PE (SSPE).

The model output was analyzed to determine whether the presence of SSPE could be predicted, although this was not part of the original RSNA PE challenge. The result is shown in Fig. 6. Classifying the presence of an SSPE using the output probabilities for presence of any PE with one threshold (maximum Youden index of 0.08) yielded 0.94 sensitivity (CI: 0.88–0.99), 0.22 specificity (CI: 0.19–0.25), an AUROC of 0.48 (CI: 0.43–0.52, p-value > 0.05), an accuracy of 76.7% and a balanced accuracy of 63.7%. Using two thresholds (0.10 and 0.95) derived from the sensitivity analysis, resulted in a maximum balanced accuracy of 0.70, a sensitivity of 0.83 and specificity of 0.57. Results for varying thresholds are shown in Figures S1. – S3. Overall, no clinically useful threshold for the discrimination of SSPEs could be derived within this analysis. Therefore, this result should be considered as exploratory only.

## Discussion

In this study, we externally validated the 2^nd^-place solution for the RSNA PE challenge with respect to the detection of the PE as well as its location (central, lobar and segmental and subsegmental) and the side of PE (right/left/bilateral). The presence of PE could be predicted with a high AUROC with no central PE being missed by the algorithm. The comparison of central versus peripheral PE showed a superiority of the model in favor of the central PE. SSPE could not be detected with sufficient accuracy by the model.

PE is a potentially life-threatening disease, making its accurate and prompt diagnosis based on CTPAs crucial for the patient’s survival^1,2^. Ongoing advancements in the optimization of AI algorithms have increasingly drawn research interest, with the long-term objective of integrating these technologies into clinical routine. Next to the training of the model, an external validation proving the model’s generalizability and thus the absence of overfitting is an important step towards a clinical implementation of AI based algorithms, which was shown by Yu et al.^8^, who reported a substantial drop of AUROC values when externally validating published models. The recent RSNA challenge to develop a DL model to predict PE on CTPA further emphasizes the value of AI in the detection of PE. Since the RSNA PE challenge winning solution has already been successfully validated externally for its performance in predicting the presence of a PE^3^, the aim of our study was to perform a detailed external validation of the 2^nd^-place DL model of Pan^11^, analyzing multiple model outcomes.

Overall, our external validation showed higher AUROC values compared to Pan when predicting the presence of PE (0.94 vs. 0.89). Our analysis also revealed a high sensitivity, specificity and accuracy of 0.80, 0.97 and 0.89, respectively. Unfortunately, these results could not be compared to Pan, since these metrics were not reported. A subgroup analysis of the AUROCs also showed higher values for central PEs (0.99 vs. 0.96), right-sided (0.95 vs. 0.91) and left-sided PEs (0.92 vs. 0.90) in favor of our analysis. The corresponding values for sensitivity, specificity and accuracy were also high. Additionally, we evaluated the model’s performance for predicting peripheral PE, where a decrease compared to the central PE was detected considering the AUROC (0.99 vs. 0.74), sensitivity (0.94 vs. 0.77), specificity (0.97 vs. 0.75) and accuracy (0.96 vs. 0.76). Using different clinical prevalences, the model performed better with lower prevalences in terms of sensitivity and accuracy, AUROC values remained highly similar, whereas a slight decrease on specificity was observed for lower prevalences. Our results are largely in line with those of Huang et al.^15^ who also demonstrated, that their model achieved similar or better results in terms of sensitivity, specificity, accuracy and AUROC using real prevalences of 14–22%.

Langius-Wiffen et al.^3^ externally validated the RSNA PE challenge winning DL algorithm. They could also show high AUROC values up to 0.96, sensitivity of 0.85 and specificity of 0.94, which is in line with our results. In comparison to our study, they only evaluated the predictions for presence of PE and did not assess any remaining model output. In terms of the general diagnostic performance of DL algorithms on detecting PE on CTPAs, a meta-analysis of five different DL algorithms by Soffer et al.^4^ showed a high pooled sensitivity and specificity of 0.88 and 0.86, respectively. Additionally, the commercially available DL algorithm Aidoc (Aidoc Medical, Tel Aviv, Israel) was tested by multiple groups, identifying sensitivities of 63.0% to 96.8% and high specificities of 95.0% to 99.9%^3,16–19^. Another algorithm cleared by the Food and Drug Administration (FDA) and tested by Ayobi et al.^20^ found a high sensitivity and specificity of 93.9% and 94.8%, respectively. This shows that open-source algorithms are able to detect PE on CTPA with similar diagnostic accuracy in terms of sensitivity and specificity as commercially available FDA-cleared algorithms. Furthermore, our analysis demonstrates that the model by Pan maintains a high level of predictive accuracy on external data, thereby supporting the advancement towards clinical implementation. In the context of a prospective study design, the model could be further evaluated under real-world conditions to assess the impact of AI-assisted radiological reporting in clinical care. The primary focus should be on its potential to reduce reporting time, improve diagnostic accuracy, and increase radiologists’ confidence across large-scale populations. In addition, further research should investigate its potential to reduce radiologists’ workload. For AI-assisted diagnosis, this could involve prioritizing examinations with a high suspicion of PE by flagging them automatically, and thus enabling faster review by the radiologist. Alternatively, the AI could be evaluated for its ability to reliably identify patients with a low probability of PE, allowing these cases to be deprioritized in favor of PE-positive patients.

Combining multiple model output probabilities enhanced the AUROC, especially when the task involved the prediction of a multinomial target variable. In certain instances, duplication of predicted probabilities was observed when both peripheral PE and side-specific predictions were selected. Hence, combining multiple model parameters could further improve the diagnostic performance in predicting the exact PE location including the side.

A special focus of our validation was on the analysis of SSPEs. Although the clinical relevance of SSPEs for patient outcomes is not yet clearly determined, their accurate diagnosis is crucial for clinical care, as SSPE were observed to possibly lead to a higher recurrence rate compared to central PEs^10^. Our results revealed a decrease in the model’s ability to correctly predict SSPE in CTPA images. Among other factors, this may be due to the model not being explicitly trained to identify SSPEs. Weikert et al.^16^ also reported a drop in diagnostic performance when the study contained subsegmental PEs. Rothenberg et al.^18^ demonstrated that the combination of AI with radiologist assessment can lead to significantly higher sensitivity and a lower miss rate in diagnosing, among others, SSPEs, but still lower than the corresponding results for central and lobar and segmental PEs. Although Ayobi et al.^20^ defined studies with SSPEs as PE negative, the algorithm, not targeted to find SSPEs, was still able to find some SSPEs. Therefore, specific training on SSPEs using a larger cohort could substantially improve the model’s ability to predict SSPEs.

Our study has some limitations. First, the initial categorization was done based on the written radiology report. Although all positive findings and 30 negative findings were double checked by an experienced radiologist, the radiologist was aware of the initial label and the radiological report, which could have biased his decision. Second, we used a thinner slice thickness of 1 mm compared to the RSNA dataset, that consisted of studies with 2 to 3 mm slices. Due to the good model performance on our dataset, we expect that the model is not negatively affected by thinner slices, although this was not directly tested within our study. The validation of an AI algorithm tested by Ayobi et al.^20^ showed a better performance with higher sensitivity and specificity for thinner slices (≤ 1.5 mm) than for thicker slices, indicating a lower influence of slice thickness on the model, as well as a high model generalizability. Third, we did not evaluate all model outcome probabilities, such as acute or chronic PE, the RV/LV ratio or the slice level prediction, since there was no reference standard for these parameters. Fourth, the resulting thresholds, that were optimized using the maximum Youden index might differ when other prevalences are used. Therefore, clinical deployment requires re-calibration of the thresholds, also probably taking a different weighting of sensitivity and specificity into account. Fifth, the number of SSPE positive studies was relatively low in our datasets, which results in a limited statistical power regarding the model’s ability to predict SSPEs.

## Conclusion

In conclusion, the external validation of the RSNA PE challenge 2^nd^-place DL model demonstrated high diagnostic accuracy in detecting PE, as well as in the differentiation of central and lobar and segmental PE with even better results compared to the training of the model. However, predicting the presence of SSPEs based on the model output probabilities was difficult, likely due to the model not being specifically trained to detect this subtype. This indicates that further clinical research is needed to optimize the prediction for SSPEs in CTPA using DL models.

## Electronic Supplementary Material

Below is the link to the electronic supplementary material.

Supplementary Material 1

## Data Availability

The datasets used and analyzed during the current study are available from the corresponding author on reasonable request.

## Abbreviations

AUROC: Area under receiver operating characteristic curve
CI: Confidence interval
CNN: Convolutional neural network
CT: Computed tomography
CTPA: Computed tomography pulmonary angiography
DL: Deep learning
FDA: Food and Drug Administration
IQR: Interquartile range
PE: Pulmonary embolism
RSNA: Radiological Society of North America
RV/LV: Right ventricular to left ventricular size ratio
SD: Standard deviation
SSPE: Subsegmental-only pulmonary embolism
