# Supplementary material for: External validation of a top-ranked model from the RSNA pulmonary embolism detection challenge: assessment of generalizability
Source: Sci Rep. 2026 Aug 8;16:24545. doi: 10.1038/s41598-026-65551-z (PMC13452819; doi:10.1038/s41598-026-65551-z)
Supplement: Supplementary file 1 [file 41598_2026_65551_MOESM1_ESM.docx]

**Supplementary Information**

| Preva-lence [%] | AUROC  (95% CI) | P-value | Threshold  (95% CI) | Sensitivity  (95% CI) | Specificity  (95% CI) | Accuracy [%] |
| --- | --- | --- | --- | --- | --- | --- |
| 5 | 0.94 (0.87-0.98) | < 0.001 | 0.45  (0.25-0.73) | 0.84  (0.71-0.96) | 0.95  (0.85-0.99) | 94.1 |
| 10 | 0.94  (0.89-0.97) | < 0.001 | 0.45  (0.30-0.58) | 0.83  (0.73-0.92) | 0.95  (0.89-0.97) | 94.0 |
| 15 | 0.94  (0.91-0.96) | < 0.001 | 0.44  (0.34-0.55) | 0.83  (0.75-0.89) | 0.95  (0.92-0.97) | 93.3 |
| 20 | 0.94 (0.91-0.96) | < 0.001 | 0.46  (0.36-0.52) | 0.82  (0.76-0.88) | 0.96  (0.94-0.97) | 92.9 |
| 25 | 0.94 (0.92-0.95) | < 0.001 | 0.45  (0.36-0.52) | 0.82  (0.77-0.86) | 0.96  (0.94-0.97) | 92.2 |
| 50 | 0.94 (0.92-0.95) | < 0.001 | 0.50  (-)* | 0.80  (0.77-0.84) | 0.97  (0.95-0.98) | 88.7 |

**Table S1.** Results of the prediction of the presence of pulmonary embolism using area under the receiver operating characteristic curves (AUROC), sensitivity, specificity and accuracy for different prevalences. The result for 50% prevalence was copied from Table 2. *No calculation of the confidence interval (CI) due to inclusion of all patients in the dataset.

**Figure S1.** Heatmap of the balanced accuracy scores for SSPE detection using the probability for the presence of any PE together with different lower and upper threshold values.

**Figure S2.** Heatmap of the sensitivity scores for SSPE detection using the probability for the presence of any PE together with different lower and upper threshold values.

**Figure S3.** Heatmap of the specificity scores for SSPE detection using the probability for the presence of any PE together with different lower and upper threshold values.
